# Complete genome sequences of *Streptomyces* spp. isolated from disease-suppressive soils

**DOI:** 10.1186/s12864-019-6279-8

**Published:** 2019-12-19

**Authors:** Stephen C. Heinsch, Szu-Yi Hsu, Lindsey Otto-Hanson, Linda Kinkel, Michael J. Smanski

**Affiliations:** 1 Bioinformatics and Computational Biology, University of Minnesota, Twin-Cities, Saint Paul, MN 55108 USA; 2 BioTechnology Institute, University of Minnesota, Twin-Cities, Saint Paul, MN 55108 USA; 3 Department of Biochemistry, Molecular Biology, and Biophysics, University of Minnesota, Twin-Cities, Saint Paul, MN 55108 USA; 4 Department of Plant Pathology, University of Minnesota, Twin-Cities, Saint Paul, MN 55108 USA

## Abstract

**Background:**

Bacteria within the genus *Streptomyces* remain a major source of new natural product discovery and as soil inoculants in agriculture where they promote plant growth and protect from disease. Recently, *Streptomyces* spp. have been implicated as important members of naturally disease-suppressive soils. To shine more light on the ecology and evolution of disease-suppressive microbial communities, we have sequenced the genome of three *Streptomyces* strains isolated from disease-suppressive soils and compared them to previously sequenced isolates. Strains selected for sequencing had previously showed strong phenotypes in competition or signaling assays.

**Results:**

Here we present the de novo sequencing of three strains of the genus *Streptomyces* isolated from disease-suppressive soils to produce high-quality complete genomes. *Streptomyces* sp. GS93–23, *Streptomyces* sp. 3211–3, and *Streptomyces* sp. S3–4 were found to have linear chromosomes of 8.24 Mb, 8.23 Mb, and greater than 7.5 Mb, respectively. In addition, two of the strains were found to have large, linear plasmids. Each strain harbors between 26 and 38 natural product biosynthetic gene clusters, on par with previously sequenced *Streptomyces* spp. We compared these newly sequenced genomes with those of previously sequenced organisms. We see substantial natural product biosynthetic diversity between closely related strains, with the gain/loss of episomal DNA elements being a primary driver of genome evolution.

**Conclusions:**

Long read sequencing data facilitates large contig assembly for high-GC *Streptomyces* genomes. While the sample number is too small for a definitive conclusion, we do not see evidence that disease suppressive soil isolates are particularly privileged in terms of numbers of biosynthetic gene clusters. The strong sequence similarity between GS93–23 and previously isolated *Streptomyces lydicus* suggests that species recruitment may contribute to the evolution of disease-suppressive microbial communities.

## Background

Roughly one third of pre-harvest crops are lost each year worldwide due to agricultural pests and disease [1]. Ninety percent of the 2000 major diseases of the 31 principle crops in the US are caused by soil-borne pathogens [2, 3], and soil microbial communities can have a protective effect [4]. Crops are particularly susceptible to disease during their establishment period and when introduced into a new geographic location [5, 6]. With the predicted changes in agricultural land use that will accompany climate change or a shift towards crops that support biofuel production, it is important to develop innovative approaches to combat crop losses to disease.

Natural and agricultural disease-suppressive soils (DSSs) have been identified that provide long-lasting and stable protection against numerous bacterial and fungal pathogens [7]. In addition to preventing crop loss, DSSs can lower the cost of production by removing the need for pesticide application. They have been reported against many major crop pathogens, including wheat take-all disease, potato scab, and wilt on melon [8–12]. Disease-suppression is correlated with increased antagonistic or competitive capacities in one or more isolates from the soil microbial community, and this behavior can emerge in a soil following long-term monoculture [7, 13–16]. However, long-term monoculture is not an attractive management strategy to create DSSs, as it generally takes a decade or more for DSSs to emerge and there would be increased plant losses in the short-term. A better understanding of the composition and ecology of DSSs will facilitate engineering soil communities for crop protection.

Recent investigations into the mechanisms of disease suppression, including metagenomic analyses of DSSs [7, 17] and phenotypic characterization of microbial isolates [18, 19], point to the importance of natural product biosynthesis within a few privileged microbial taxa. Not only are known natural product producers, Actinomycetes and Pseudomonads, enriched in DSS samples, but interruption of natural product biosynthesis genes interferes with disease-suppression [17]. Further, ecological models that describe the emergence and maintenance of DSSs propose a link between plant biodiversity and the evolution of DSSs. In soils supporting diverse plant species, root exudates and decomposing biomass supply diverse nutrients to soil microbes, which can evolve to co-exist via niche-differentiation. However, in long-term mono-species plant plots, the abundant but non-diverse plant nutrients create a competitive soil environment that favors the evolution of antagonism through antibiosis [7].

Because the metagenomics, phenotypic, and theoretical work all point to the importance of natural products in the formation and maintenance of DSSs, we have sought to better understand natural product biosynthesis in these communities. The observation that isolates from DSSs are more likely to produce antibiotics that target sympatric isolates [20] supports several alternative hypotheses surrounding natural product biosynthesis. Highly antagonistic microbial strains should either (i) encode more natural product biosynthetic gene clusters (BGCs) in their genomes than isolates from non-suppressive soils, (ii) encode the same number but actively express a greater percentage of their BGCs, or (iii) produce the same number of natural products, but these compounds are enriched in the biological activities that are important for the formation of DSSs. The first hypothesis is directly testable through whole genome sequencing and comparison.

Here we present the first genome sequences for *Streptomyces* spp. isolated from DSSs. Genomes were sequenced with both long-read PacBio and short-read Illumina technology to produce high-quality and nearly complete sequences for each strain. Bioinformatic analyses highlight the importance of natural product biosynthesis in these isolates, and comparative genomics provides insight to the evolution and ecology of DSSs.

## Results

### Isolation and phenotypic characterization of strains

Each of the strains sequenced for this study were selected because (i) they were isolated from soils with measurable disease-suppressive characteristics, and (ii) they displayed strong phenotypes in competition or signaling assays.

*Streptomyces* sp. GS93–23 was isolated from a potato scab-suppressive plot in Grand Rapids, MN using the Anderson Air Sampler isolation method [21, 22]. This strain performed the best of ~ 800 isolated strains at combating potato scab [21]. GS93–23 also shows antifungal activity against *Phytophthora medicaginis* and *Phytophthora sojae*, two fungal pathogens of alfalfa. This activity extended to soil studies, where GS93–23 protected alfalfa, reducing the percentage of dead plants from 50 to 0% when pathogens were seeded at low density [23]. Further, compared to no-treatment controls, GS93–23 increased plant growth and yield (forage weight per pot), suggesting direct or indirect plant growth promotion activity. Lastly, GS93–23 was found to be strongly antagonistic against other *Streptomyces* spp., but did not reduce nodule production by rhizobial bacteria [23].

*Streptomyces* spp. S3–4 and 3211–3 were isolated from pathogen suppressive soils located in the Cedar Creek Ecosystem Science Reserve (CCESR), an NSF long-term ecological research site [24]. S3–4 was isolated from soil in a long-term big bluestem (*Andropogon gerardii*) monoculture plot and is antagonistic against sympatrically evolved soil isolates [25]. Strain 3211–3 was isolated from a native prairie control plot at CCESR. It has a strong signaling phenotype, defined as the ability to elicit antibiotic/antifungal production in strains with which it is cultured on close spatial proximity [26].

### PacBio sequencing and assembly of genomes

Initial genome sequencing and scaffold assembly was performed on a Pacific Biosciences (PacBio) RS single molecule sequencer (October 2014). Genomic DNA was size-selected using Blue-Pippen 20 kb and sequenced in three SMRTcells each. The first two SMRTcells for each genome were run using P4 chemistry, and third SMRTcell was run for each genome with P6 chemistry. Initial read assembly using the PacBio HGAP2 algorithm and sequence polishing using the PacBio Resequencing algorithm produced genome sizes of and contig numbers shown in Table 1. Final coverage was >100x for each genome.

**Table 1:** Comparison of general chromosome characteristics

	GS93–23	3211–3	S3–4
Assembled genome size (bp)	8,243,179	8,991,292	8,056,350
Chromosome size (bp)	8,243,179	8,232,231	> 7,504,752
Chromosome topology	Linear	Linear	Linear
Chromosome G + C content	72%	71%	73%
rRNA operons	7	7	8
tRNA genes	66	77	73
Protein-coding genes	7188	8087	7071
Natural product BGCs	26	38	28

The high GC-content of *Streptomyces* genomes produces many homopolymer G and C stretches, which can produce errors during base-calling and genome assembly. Low-coverage Illumina sequence data was collected for error correction. Illumina sequencing was performed on a Mi-seq instrument to collect 2 × 250 base paired end reads equating to 110-fold (3211–3), 118-fold (GS93–23), or 155-fold (S3–4) coverage for each genome. Final, error-corrected genome sequences were generated by mapping Illumina short reads to PacBio-generated reference genomes using the BreSeq algorithm [27], and incorporating single nucleotide polymorphisms (SNPs) and short Indels using the Pilon algorithm [28].

### Comparison of Illumina-corrected and PacBio-alone genome sequences

The short-read corrected genome sequences were compared to the PacBio-only assemblies, and 70, 295, and 335 SNP/Indels were present between the two assemblies for GS93–23, S3–4, and 3211–3, respectively. In each case, the vast majority were single base insertions in homopolymer stretches. We next sought to verify that the short-read corrected sequences were indeed a better representation of the actual genome sequence, as the two sequencing platforms are known to generate different types of errors. To determine which sequence variant was correct for each SNP/indel, translated protein sequences at each of the 295 SNP/indel loci in the S3–4 genome were compared against the NCBI GenBank non-redundant database, with the assumption that a frameshift resulting from an indel will result in a worse top blast hit for a stretch of DNA. Additional file 1: Figure S1 shows the comparison of significance score for BLASTx results of searching a fragment of DNA +/− 150 bases from the variant loci. This analysis is only expected to reveal the correct sequence variant when (i) the indel is present within a coding DNA sequence (CDS), (ii) correct protein sequences for close homologs are present in GenBank, and (iii) the 300 basepair window that is searched is sufficiently focused such that top BLAST hits align to the translated query in the region of the variant locus (i.e. at the center of the query, not the edges). We find that the Illumina-corrected sequence returns a top BLASTx hit with lower (better) E-value twice as often as the uncorrected sequence. The average E-values for the top BLASTx hit alignment are six orders of magnitude lower (better) for the short-read corrected sequences compared to the PacBio-only sequences. Because of this, we use the short-read corrected genome sequences for the remaining analyses.

### General characteristics of the genome sequences

**Fig. 1:**
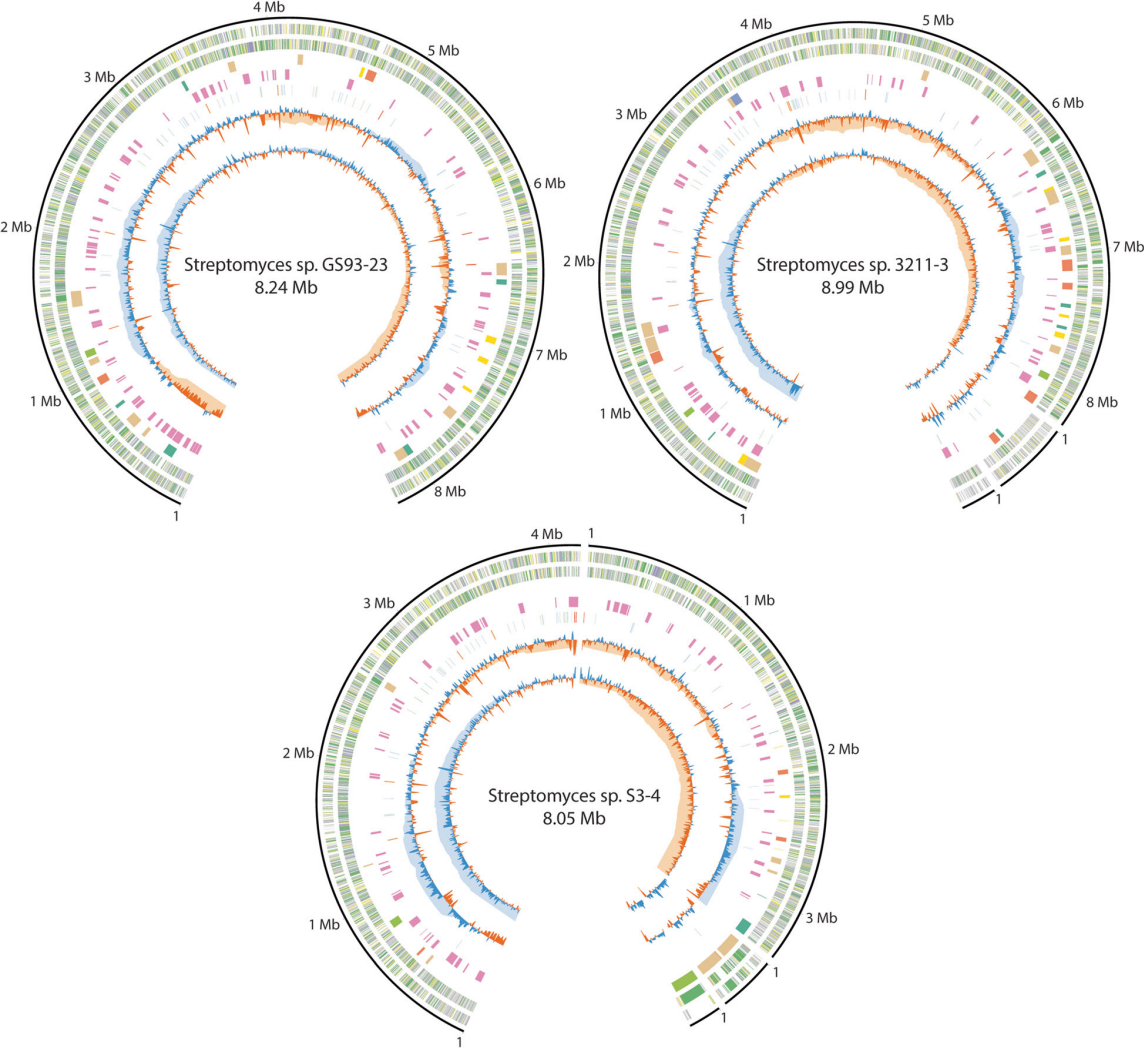
Schematic representation of genome sequences for strains GS93–23, 3211–3, and S3–4. Outer, solid black ring shows contig length in Mb. Second and third rings show annotated CDSs in the forward or reverse orientation, respectively, colored by functional classification. Genes involved in metabolism are green, information storage and processing are purple, cellular processes and signaling are yellow, and unknown functions are grey (see Table 2). Fourth and fifth rings show high-confidence and putative natural product BGCs, respectively. High-confidence BGCs are colored by biosynthetic class, with polyketides light green, non-ribosomal peptides orange, terpenes yellow, nucleosides purple, RIPPs dark green, and hybrid clusters tan. Sixth ring shows functional RNA elements, including rRNA (reverse orientation orange, forward orientation red) and tRNAs (reverse orientation blue, forward orientation green). Seventh and eighth rings show G + C content and G + C skew, respectively. Each is shown for two window sizes: 10 Kb (dark blue above average, dark orange below average) and 1 Mb (light blue above average, light orange below average). Only the 10 Kb resolution data is shown for plasmids

We were able to assemble the chromosome as a single large contig for strains GS93–23 (8.24 Mb) and 3211–3 (8.23 Mb), and as two large contigs for S3–4 (4.19 Mb and 3.31 Mb) (Fig. 1 and Table 1). For S3–4, the two chromosome arms can be oriented relative to one other with high confidence based on GC-skew, orientation of rRNA operons, and enrichment of specialized metabolite gene clusters at chromosome arms (Fig. 1, rings 8, 6, and 4, respectively). Manual attempts to close the gap by retrieving PacBio reads that mapped to each contig were unsuccessful. The gap is present in a locus that is especially repetitive, with 3 rRNA operons in close proximity. The overall G + C content (71–73%) and differences in G/C skew for the chromosome arms in each genome are similar to what has been reported for other genomes from this genus [29–34]. In addition to the large linear chromosomes, strains 3211–3 and S3–4 each contain two large linear plasmids (519 Kb and 240 Kb for 3211–3, 349 Kb and 203 Kb for S3–4).

Annotation of the genomes with the Prokka software tool [35] identified 7188 CDSs, 7 ribosomal RNA operons, and 66 tRNAs for GS93–23. Similar numbers of annotated genes were present in the S3–4 genome (7071 CDSs, 8 rRNA operons, 73 tRNAs), and slightly more in the 3211–3 genome (8087 CDSs, 7 rRNA operons, 77 tRNAs), accounting for its larger total size. Gene products were assigned to Clusters of Orthologous Groups (COGs) using the BASys platform [36]. Functional categorization of proteins reported in Table 2 in comparison to the model organism, *S. coelicolor* A3 (2) were performed with EggNOG-mapper [37].

**Table 2:** COG functional categories

COG	GS93–23		3211–3		S3–4		*S. coelicolor*	
	%	Num.	%	Num.	%	Num.	%	Num.
**Cellular processes and signaling**								
Cell division and cytoskeleton	0.5	38	0.5	40	0.5	38	0.5	38
Defense mechanisms	1.4	106	1.3	112	1.2	90	1.4	115
Signal transduction mechanisms	4.5	335	4.5	394	4.4	328	4.6	385
Cell wall/membrane/envelope biogenesis	2.9	217	2.6	228	2.8	209	2.8	235
Secretion	0.5	34	0.5	40	0.5	34	0.5	43
Posttranslational modification	2.0	151	2.1	186	2.1	154	1.9	158
**Information storage and processing**								
Translation, ribosomal structure and biogenesis	2.4	180	2.1	182	2.5	188	2.3	192
Transcription and RNA processing	9.4	708	7.6	666	8.1	597	9.4	786
Replication, recombination and repair	2.7	204	6.3	551	4.0	295	3.8	318
**Metabolism**								
Energy production and conversion	4.7	353	3.7	330	4.3	316	4.5	374
Carbohydrate transport and metabolism	5.2	392	3.7	325	4.2	308	6.1	509
Amino acid transport and metabolism	5.9	439	4.5	393	5.1	376	4.7	395
Nucleotide transport and metabolism	1.6	120	1.2	106	1.4	106	1.2	103
Coenzyme transport and metabolism	2.0	147	1.7	148	2.0	146	1.7	143
Lipid transport and metabolism	2.8	207	2.7	240	2.7	199	2.4	199
Inorganic ion transport and metabolism	3.5	261	3.2	280	3.2	237	4.0	335
Secondary metabolism	2.5	189	2.2	194	2.8	209	2.0	168
**Poorly characterized**								
Function unknown	30.9	2314	30.1	2658	32.2	2386	30.8	2564
No COG in database	14.7	1102	19.8	1743	16.2	1198	15.2	1265

### Annotation of natural product biosynthetic gene clusters

Because natural product biosynthesis is thought to play a mechanistic role that underpins the ecology of disease suppressive soils [17, 38], we have analyzed the genomes for their biosynthetic potential using the antiSMASH 3.0 toolkit [39]. We conservatively assigned specific molecules to these BGCs only when the annotated gene clusters share 100% of the biosynthetic genes from previously characterized BGCs by manual comparison (Additional file Information). For ribosomally produced and post-translationally modified peptides (RiPPs), we predict the production of minor structural variants when the sequence of precursor peptides is slightly different than in characterized BGCs. The 26 high-confidence BGCs identified in the GS93–23 genome include known pathways for RiPP cyclothiazomycin [40], the dienoyltetramic acid streptolydigin [41], and the lipoglycopeptide mannopeptimycin [42]. The 38 high-confidence BGCs in the 3211–3 genome include known pathways for the chlorinated non-ribosomal peptide tambromycin [43], the siderophore coelichelin [44], and terpenoid 2-methylisoborneol [45]. The 28 high-confidence BGCs in the S3–4 genome include known pathways for 2-methylisoborneol, and the aminoglycoside streptothricin [46]. In addition, all three genomes contain the highly conserved BGCs for the siderophore desferrioxamine b [47], terpenes geosmin [48] and hopene [49], minor structural variants of lantibiotic SapB [50], and osmoprotectant ectoine [51].

The majority of BGCs identified in these genomes remain uncharacterized. Intriguing pathways include a 178 Kb polyketide cluster on a plasmid in S3–4 that putatively encodes a 60-member macrolide, and a pyrrolopyrrole-containing metabolite in 3211–3.

### Comparison to closest sequenced relatives

**Fig. 2:**
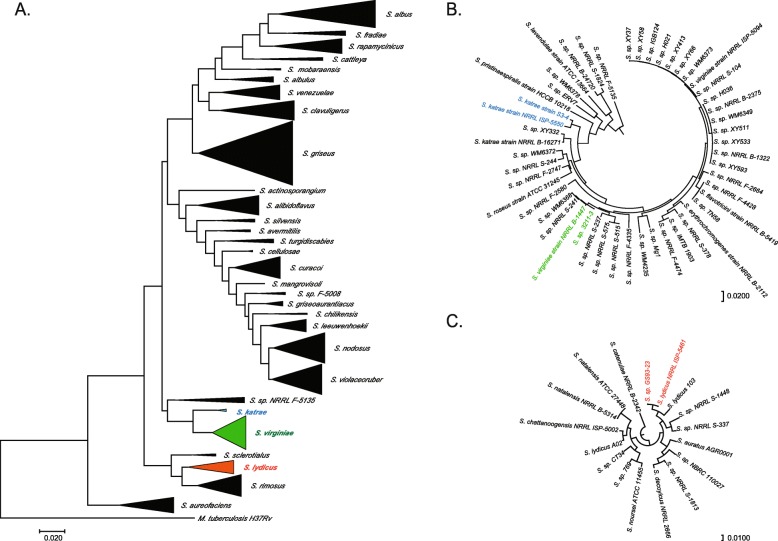
Molecular phylogeny of newly sequenced strains. **(a)** Phylogenetic tree of 496 publicly available *Streptomyces* genomes. *Mycobacterium tuberculosis* H37Rv was used as outgroup. Select regions of the *atpD, gyrB, recA, rpoB,* and *trpB* genes were concatenated and used to generate a multi-locus alignment in the MEGA7 software package. Genetic distances (average nucleotide identity) generated from the multisequence alignment were used to build a phylogenetic tree using the maximum likelihood method. Clades containing the newly sequenced genomes are *S. katrae (*S3–4, blue), *S. virginiae* (3211–3, green), and *S. lydicus* (GS93–23, red). Subtrees composed of *S. katrae* and *S. virginiae* **(b)**, and *S. lydicus* **(c)** showing the newly sequenced isolates and their closest relatives

We compared the draft genome sequences to a collection of 500 publicly available actinomycete genomes using multi-locus sequence comparison to identify the closest sequenced relative of each (Fig. 2). S3–4 groups with the small *Streptomyces katrae* clade near type strain NRRL-ISP 5550 [52]. Strain 3211–3 is in the neighboring *Streptomyces virginiae* clade defined by the type strain NRRL ISP-5094 [53]. GS93–23 clusters with the *Streptomyces lydicus* type strain NRRL-ISP 5461 [54].

**Fig. 3:**
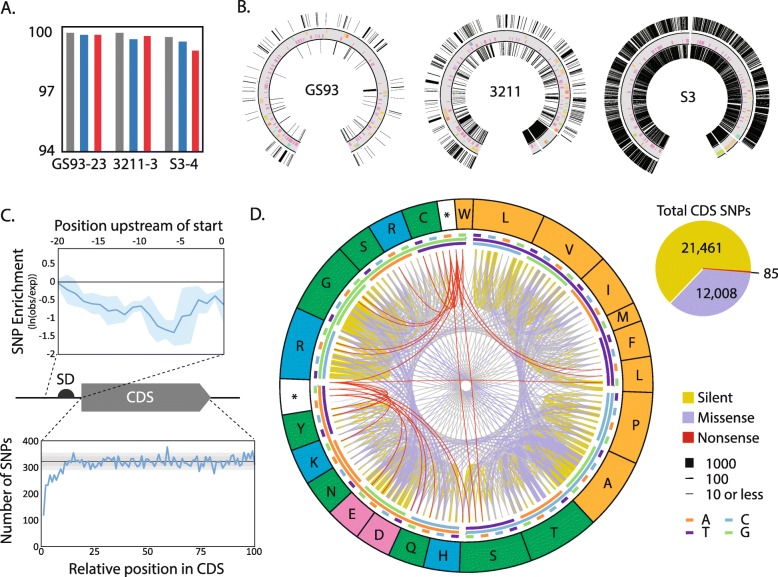
Comparative analysis of with closest sequenced relatives. **(a)** Sequence identity between newly sequenced strains and closest relatives, with 16S rDNA (black), *rpoB* gene (dark grey) and multilocus sequence comparison (light grey) shown for each pair of strains. **(b)** Genomic location of alignment gaps larger than 100 bp. Grey ring represents newly sequenced genomes, with high-confidence and putative BGCs labeled as in Fig. 1. Outer ring shows location of extra sequence present in closest relative but missing in our newly sequenced strain. Inner ring shows location of extra sequence present in newly sequenced strains but missing from closest relative. **(c)** SNP analysis of strain GS93–23 and its closest relative ISP-5461. Pie-chart in upper right shows relative proportion of silent, missense, and nonsense mutations. Circle chart at left shows frequency of all SNPs found in CDSs, with the outer ring showing one-letter code for amino acids, colored according to chemical property (hydrophobic, orange; hydrophilic, green; basic, blue; acidic, pink). Three-base code is shown using three inner rings, with innermost representing the first codon position and outermost representing the last codon position. For each codon, there are two nodes on the graph. In the clockwise direction, the first node corresponds to a codon in GS93–23 and the second node to ISP-5461. Each CDS SNP is represented by an arc connecting a codon in GS93–23 to a codon in ISP-5461, with the width of the arc indicating number of instances of that mutation. **(d)** Location of SNPs relative to CDS position. Top line graph shows enrichment of SNPs upstream of start codons using absolute positions, with the solid blue line showing average value for a sliding 3-base window and the light-blue filled region showing one standard deviation in either direction. Bottom line graph shows SNP abundance versus relative position in CDS, where relative position equals absolute position divided by CDS length. Black line and grey boxes show average SNP abundance and 1-, 2-standard deviations as calculated for the last 90% of the CDS. CDS: coding DNA sequence; SD: Shine-Dalgarno sequence

We identified closely related genomes in the available whole-genome sequence databases for each of our DSS isolates (Fig. 3). For each of our newly sequenced strains, a previously published genome was available with high sequence similarity in several common phylogenetic markers (16S rRNA, *rpoB*, and multi-locus sequencing (MLS) using ribosomal proteins) (Fig. 3a). Our closest pair of new and previously reported genomes is GS93–23 and *S. lydicus* NRRL ISP-5461, which share 100% identity of 16S rRNA and 99.92% identity using MLS comparison. Even our most divergent pair, S3–4 to *Streptomyces* sp. WM6372, shared > 98% identity at the 16S rRNA level and > 96% identity at the *rpoB* level, and 93.72% by four-gene MLS comparison (*atpD, gyrB, rpoB, trpB)*.

Genome pairs were compared to determine the amount of shared sequence across the entire genome (Fig. 3b). Alignments were constructed in Mauve and alignment gaps were mapped back to the new high-quality reference genomes. Alignment gaps between of GS93–23 and ISP-5461 are uniformly distributed across the chromosome. Insertions or deletion events greater than 100 bp account for only 4.5% of the genome sequence as a whole (Fig. 3b), with a similar proportion being lost/gained in BGCs as in the rest of the genome (Fig. 3b).

The high-level of sequence conservation between GS93–23 and ISP-5461 allowed us to examine the micro-scale evolution of these genomes. There are approximately 40,000 SNPs between the two, making the sequence identity in the aligning sequences greater than 99.5%. Interestingly, the position of SNPs relative to CDSs shows a marked de-enrichment in (i) the approximate position of the Shine-Dalgarno sequence in the 5′-UTR, and (ii) the 5′ end of the CDS (Fig. 3c). This suggests a selection for maintaining relative translation rates of encoded genes, as both loci are important in determining translation initiation rates in bacteria [55]. Most of the ~ 33,000 SNPs in CDSs encode silent mutations. Of the missense mutations, the majority are conservative in terms of amino acid chemistry (Fig. 3d). The ratio of synonymous to non-synonymous mutations (*d*_*S*_*/d*_*N*_) is 1.8, which is substantially lower than seen in housekeeping genes in *E. coli* and invasion genes from *S. enterica* [56, 57], suggesting that there has been little selective pressure against non-synonymous mutations and that these two strains belong to the same clonal complex [58, 59].

Despite the strong similarity between GS93–23 and ISP-5461, there are still substantial differences between the two strains. GS93–23 contains 98 genes that are missing in ISP-5461, and ISP-5461 contains 11 unique genes. 66/98 genes unique to GS93–23 are of unknown function. Of genes with functional annotations the largest categories specific to GS93–23 are transcriptional regulators (11/98) and metabolic enzymes (10/98). Of the genes unique to ISP-5461, only a single gene was of unknown function. The largest functional categories for genes unique to ISP-5461 also were transcriptional regulators (3/11) and metabolic enzymes (3/11).

The other two DSS genomes presented here are more divergent from the nearest sequenced relative. Both 3211–3 and S3–4 have two large plasmids that are absent in their closest relatives, *S. virginiae* NRRL B-1447 and *S. katrae* NRRL ISP-5550, respectively. These changes alone account for 9 and 7% of the total genome content, respectively. The plasmids in S3–4 are rich in secondary metabolism genes, with four large gene clusters totaling roughly 500 kb of sequence. Besides the plasmid differences, the chromosome of 3211–3 contains 285 large (> 100 bp) insertions compared to B-1447, totaling 609 kb of new sequence, and 309 large deletions totaling 758 kb of sequence lost. In the regions that do align, there are 102,000 SNPs, corresponding to an average sequence identity of 98.7% across the genome. The S3–4 genome lacks a close homolog in the sequence databases. Despite sharing 96.3% sequence identity of the *rpoB* gene, 26% of the S3–4 genome does not align with the WM6372 sequence.

We next compared the natural product biosynthetic potential for these three strains by analyzing their BGC content. Our closest pair, GS93–23 and ISP-5461, share 26/26 of the high-confidence BGCs and 61/64 ‘putative’ clusters (co-localized clusters of genes that belong to COGs typically found in BGCs, but which lack canonical secondary metabolism signature sequences). The next closest pair, 3211–3 and B-1447, which share 99.7% similarity of the *rpoB* gene, have in common only 31/38 of the high-confidence BGC annotations, which is driven mostly by the presence of two plasmids in 3211–3 missing from B-1447. Between S3–4 and WM6372 (96.3% identity of *rpoB*), 12/28 of high-confidence BGCs are shared, and 27/54 ‘putative’ clusters. These relationships between genetic distance and BGC overlap follow the general trend for *rpoB* conservation and non-ribosomal peptide synthetase (NRPS) BGC overlap described by Doroghazi et al. [60].

### Signaling potential analysis

One possible organization for a highly antagonistic microbial community would have a keystone species that produces a signal to induce antibiotic production in many other community members. The University of Minnesota DSS strain library was assayed for signaling potential using a plate-based phenotypic assay [26] (Kinkel, unpublished data). Strain 3211–3 was selected for whole genome sequencing because it is among the best signalers of antibiosis in our library of DSS isolates. The signaling assay requires dilution of a signaling molecule through solid agar medium, so signaling through cell-cell contact can be ruled out as a mechanism. We looked for genomic features that could explain the signaling promiscuity in 3211–3.

Signaling between *Streptomyces* can be mediated by several well-known classes of hormone-like signaling molecules [61] including γ-butyrolactones [62], furans [63], γ-butenolides [64], SapB [65] -like RiPPs, diamino-bis(hydroxymethyl)-butanediol [66], and diketopiperazines [67]. Signaling can also be mediated by sub-inhibitory concentrations of antibiotics [68–70]. We first looked for the presence of BGCs encoding hormone-like signaling molecules in 3211–3. There are two γ-butyrolactone BGCs in this genome and a SapB BGC, but this number is comparable to other sequenced *Streptomyces*. There is no evidence that 3211–3 produces an unusually diverse set of hormone-like signaling molecules.

**Fig. 4:**
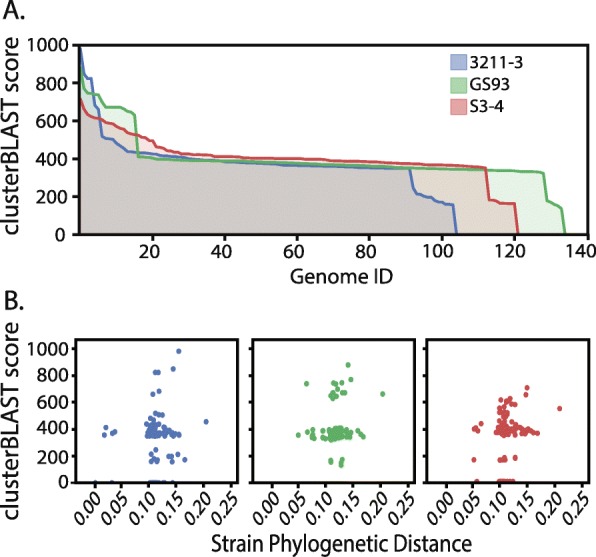
Signaling potential analysis of newly sequenced strains. **(a)** Distribution of homologous gamma-butyrolactone biosynthetic gene clusters throughout 496 *Streptomyces* genomes. Area plots show clusterBLAST scores for 3211–3 (blue), GS93–23 (green), S3–4 (red). Genome ID’s for hits have been sorted in order of 3211–3. **(b)** Relationship between genetic distance and clusterBLAST score. Genetic distances between the three newly sequenced isolates and the genome hits from the signaling potential MultiGeneBLAST analysis genomes were obtained by multilocus alignment of the *atpD, gyrB, recA, rpoB,* and *trpB* genes

A second possibility is that 3211–3 does not produce many diverse hormone-like signaling molecules, but the molecule they do produce can be sensed by many species of *Streptomyces*. There are at least fifteen unique γ-butyrolactone signals produced by the genus, and unfortunately it is not possible to predict the specific γ-butyrolactone chemical structure from sequence information alone. However, we reasoned γ-butyrolactone biosynthesis genes and receptors that produce/sense the same compound will have a higher degree of sequence similarity than those producing/sensing different compounds (i.e. functionally similar gene clusters would share greater sequence similarity), as this gene cluster does not closely correlate with other phylogeny (Fig. 4). We performed a CLUSTER-BLAST analysis with the γ-butyrolactone biosynthesis protein ScbA and the receptor AfsR against the set of sequenced *Streptomyces* genomes. Again, we did not see any evidence that 3211–3 produces a more widely-sensed hormone-like signaling molecule.

A third possibility is that 3211–3 is a prolific signaler due to production of sub-inhibitory concentrations of antibiotics (SICA). This genome encodes more ‘high-confidence’ BGCs than the two genomes from strongly antagonistic DSS isolates (Table 1)*.* Among 125 complete *Streptomyces* genomes with antiSMASH 4.1 (Additional file 1: Table S4), the number of high-confidence BGCs in 3211–3 places it in the top 16% in terms of BGC content. Since there is no clear genomic signature that allows us to explain the signaling potential in 3211–3, teasing apart its ability to elicit antibiosis in so many diverse isolates will require future molecular genetic experiments.

## Discussion

Bacteria within the genus *Streptomyces* are ubiquitous in terrestrial soils and marine sediments and have garnered much attention for their ability to produce medicinal natural products. The past decade and a half of genome sequencing efforts [30, 60, 71] revealed that the majority of natural products encoded in the genomes of *Streptomyces* spp. remain undiscovered and have reinvigorated natural product discovery via genome mining [72, 73]. Most genomes deposited in public sequence databases have been sequenced using Illumina short-read technology. The large size, repetitive nature, and high G + C content of *Streptomyces* genomes makes them difficult to fully assemble from short reads, and so roughly 90% of the available genomes are only available in draft status; typically hundreds of contigs with an average N50 of thousands of bases. With a combination of PacBio and Illumina sequence data, we were able to assemble high-quality genome sequences where the > 8 Mb chromosome assembles as a single contig in two strains and as two contigs in the third.

We initially predicted that the increase of genome quality would correspond to an improved ability to identify BGCs that would have been broken up between many small contigs in a short-read only assembly. However, the difference in quality does not appear to effect estimations of natural product biosynthetic potential. For example, in *S. lydicus* ISP-5461, 26 of the 26 high-confidence BGCs found in GS93–23 were also predicted using the short-read only assembly contigs.

One advantage to generating single-contig genomes using long-read data is the ability to map the chromosomal location of BGCs. In order to help prioritize isolated *Streptomyces* strains for whole-genome sequencing, there have been previous attempts to correlate sequence conservation of phylogenetic markers with BGC conservation between two or more genomes [60]. After sequencing 1000 actinomycete genomes, Metcalf et al. found that a 99% sequence identity between concatenated ribosomal protein sequences correlates with a 73 and 80% conservation of Type I polyketide synthase (PKS) and NRPS clusters, respectively [60]. Our data supports the rapid diversification of secondary metabolite gene clusters, and suggests that this is primarily driven by changes in episomal elements, not by changes to the core genome. This information could make future sequencing campaigns more efficient by limiting sequencing efforts in closely related strains to isolated plasmids.

Bacterial genome organization has been described as mosaic [74–76], referring to the composition of a vertically-inherited (clonally-expanded) backbone interspersed with laterally-transferred mobile elements. Mutations accumulate in clonal complexes between bouts of periodic selection [59]. The genomic comparison of GS93–23 and ISP-5461 suggests that these strains are part of the same clonal complex, despite being isolated 850 km apart and several decades removed. Our analysis of the SNP accumulation in relationship to relative location within genes shows a de-enrichment of sequence variation in regions known to control translation initiation rates. This points to a microevolution of genomes where there is a selection to maintain relative expression levels of genes during clonal expansion. We have previously shown that transfer of multi-gene systems between hosts from the same genus can result in wildly different relative expression levels [77]. These likely result from the accumulation of subtle differences between the transcription/translation machinery and corresponding cis-acting regulatory elements that co-evolve during clonal expansion. Taken together, the importance of maintaining relative expression levels during microevolution and the changes between seemingly closely related species likely contributes to low success rates and low titers during heterologous introduction of BGCs to model host strains [78].

We sequenced the three strains presented here in hopes to gain insight towards the mechanisms and ecology that underlie DSSs. While the sample size is small, there is no indication that the increased antibiosis observed in DSS isolates compared to isolates from non-suppressive soils is due to an increased number of BGCs. Transcriptomic and chemical characterization of these and other DSS isolates is pending. With over 500 species of *Streptomyces* currently recognized [79] and roughly 800 draft *Streptomyces* genomes available in public databases at the time of this study, we were initially surprised by the level of sequence conservation between these strains and previously sequenced genomes. The level of divergence between GS93–23 and ISP-5461 is only ten times greater than clonally-related lab-cultivated strains of *E. coli* separated by only 20 years of evolution [80]. There are a few possible explanations for this. First, species groups are not expected to be equally abundant. It is likely that the genomes already present in the public databases are those of highly abundant clonal complexes. The similarity between these genomes and extant sequences reflects the fact that no attempts were made to bias our strain selection towards rare *Streptomyces*. A second possibility is that the ecology of DSSs has selected for strains that are also abundant in sequenced collections. This makes sense in light of the experimental data and ecological models that suggest DSSs community members are selected for their antagonistic phenotypes [7]. Likewise, most *Streptomyces* strains whose genomes are in public databases were originally isolated and maintained in collections of drug discovery groups. If this is true, it will suggest that evolution of DSS isolates occurs on the level of the genome/strain, not the individual genes, contrary to what has been observed in other environments [81]. Strain recruitment is a proposed mechanism of the establishment of disease suppressive soils [82], in which plants support the maintenance of those microbial strains which inhibit phytopathogens. 16S sequencing and denaturing gel electrophoresis of the rhizosphere microbiome of strawberry plants showed that the Actinobacteria community profile was more similar between species of strawberry plant, regardless of site, when compared to oil rape rhizosphere communities [83]. It is not unreasonable, then, to assume that under the dispersal-recruitment model, that ancestral bacterial strains that were beneficial to plant growth would be under similar selective pressures if co-evolving with the same plant species in distant locations.

## Conclusion

In summary, we have added three high-quality whole genome sequences to the growing number of sequenced *Streptomyces* isolates. Each genome is rich with yet-uncharacterized natural product biosynthetic potential. While genome sequence alone was not sufficient to explain the observed phenotypes of DSS isolates, it is an important first step to future investigations of gene expression and function.

## Methods

### Preparation of high molecular-weight DNA

The three strains of *Streptomyces* sequenced for this study were obtained from a culture collection maintained by Linda Kinkel at the University of Minnesota. Single colonies are isolated on IWL-4 solid medium and used to inoculate 4 mL liquid cultures in R2YE medium. Following three days of growth, cells are harvested by centrifugation and washed with a 10% sucrose solution. Mycelia are resuspended in 450 μL TSE buffer (15% sucrose, 25 mM Tris, 25 mM EDTA, pH 8) with 5 mg/mL lysozyme and incubated at 37 °C for one hour. Cells are lysed by addition of 225 μL of 2% SDS over a 5 min room temperature incubation. Following a phenol:chloroform extraction (100 μL neutral phenol, 50 μL chloroform), supernatant is transferred to a tube containing 60 μL 3 M sodium acetate and 700 μL isopropanol to precipitate gDNA. DNA is pelleted by centrifugation and resuspended in 500 μL TE buffer (10 mM Tris, 1 mM EDTA, pH 8). To remove RNA, 10 μL RNase (10 mg/ml) is added to the sample and incubated at room temperature for at least 15 min. Next, a second phenol:chloroform extraction (300 μL neutral phenol, 150 μL chloroform) is performed followed by a final extraction with 300 μL chloroform to remove trace phenol. DNA in the supernatant is precipitated with 50 μL 3 M sodium acetate and 350 μL isopropanol and incubated on ice for 30 min. Final gDNA is resuspended in 150 μL TE buffer and quality is assessed by agarose gel electrophoresis, spectrophotometry, and PicoGreen analysis.

### DNA sequencing and assembly

We performed PacBio long-read sequencing using protocols for 20 Kb insert size with BluePippin Size Selection (Saga Science). For each of the three genomic DNA samples, sequencing was performed using P4 chemistry on two SMRT cells and using P6 chemistry on an additional SMRT cell from November 2014 to January 2015. In total, subread filtering from the three SMRT cells yielded 1.26 Gb (S3–4), 1.40 Gb (GS93–23), and 1.18 Gb (3211–3) of sequence data with average read lengths of 6703 kb, 6782 kb, 6478 kb, respectively and *N*_50_ values of 9095 kb, 8819 kb, and 8680 kb, respectively.

### Short-read sequencing and error correction

Illumina MiSeq sequencing was performed at the UMN Genomics center in March 2015. The three genomic DNA samples were uniquely barcoded and sequenced alongside genomes from unrelated bacteria to account for 30% of a MiSeq lane. Nextera library prep was performed using standard protocols at the University of Minnesota Genomics Center. The 250 nt paired-end reads were mapped to the PacBio-reference genome sequence using Breseq [27] to generate. BAM files. Single-base differences and small indels were corrected using Pilon to generate the final error-corrected genome assembly.

### Annotation of genomic features

Prokka [35] is a command line software tool that uses Prodigal [84] for coding DNA sequence (CDS) annotation, RNAmmer [85] for ribosomal RNA annotation, Aragorn [86] for transfer RNA annotation, SignalP [87] for signal leader peptide annotation, and Infernal [88] for non-coding RNA annotation. Each genome was annotated with the Prokka software package using default options and the ‘--compliant’ command to force compliance with GenBank.

Assignment of putative functional categories to CDSs was performed using the BASys [36] web server (https://www.basys.ca/). For each CDS, start position, end position, strand information, and a unique identifier was provided in tabular format to ensure that Prokka-generated annotations would be used for clusters of orthologous genes (COG) assignment in place of the default Glimmer algorithm. The following options were selected for functional assignment by BASys: Gram positive, Linear contig, Bacterial genetic code. Functional assignments of proteins in Table 2 were performed with EggNOG-mapper [37]. The following EggNOG-mapper settings were selected: mapping mode was set to DIAMOND [89], taxonomic scope was set to all bacteria, all orthologs were used, and non-electronic gene ontology evidence terms were selected.

### Phylogenetic analysis

*Streptomyces* genomes were obtained from PATRIC (https://www.patricbrc.org/). Nucleotide sequences for molecular phylogeny markers *atpD, gyrB, recA, rpoB,* and *trpB* were extracted. Regions for comparison were identified and concatenated head-to-tail in-frame [90, 91]. Multi-sequence alignment of concatenations, and maximum-likelihood tree construction was performed in MEGA7 [92]. For the S3–4 subtree phylogeny the *recA* sequence was not available for WM6372 and a four-gene concatenation was used.

## Additional file

**Additional file 1: Table S1.** *Streptomyces sp.* GS93–23, **Table S2.** *Streptomyces sp.* 3211–3,**Table S3.** *Streptomyces sp.* S3–4 gene clusters, **Table S4.** Cluster abundance for 125 Complete *Streptomyces* genomes, **Figure S1.** Indel comparison of Illumina polished vs. PacBio only assemblies.

## Data Availability

The genome sequences reported here are available in GenBank under the accession numbers NZ_CP020042 for *Streptomyces* sp. S3–4, NZ_CP020039 for *Streptomyces* sp. 3211–3, NZ_CP019457 for *Streptomyces* sp. GS93–23.^93^ For the NCBI submitted S3–4 genome, the two large chromosomal contigs were joined together by 100 ambiguous bases. The second half of the chromosome starts at 41915460 bp.

## Abbreviations

BGC: biosynthetic gene cluster
BLAST: basic local alignment search tool
CCESR: Cedar Creek Ecosystem Science Reserve
CDS: coding sequence
COG: clusters of orthologous groups
DNA: deoxyribonucleic acid
DSS: disease-suppresive soil
gDNA: genomic deoxyribonucleid acid
HGAP2: Hierarchical Genome Assembly Process
InDel: insertion-deletion
Kb: kilobase
km: kilometer
Mb: megabase
MLS: multi-locus sequence
NCBI: National Center for Biotechnology Information
nr: non-redundant
NRPS: nonribosomal peptide synthetase
NRRL: Northern Regional Research Laboratory
PacBio: Pacific Biosciences
PKS: polyketide synthase
RiPP: ribosomally synthesized and post-translationally modified peptides
RNA: ribonucleic acid
RNase: ribonuclease
rRNA: ribosomal ribonucleic acid
SICA: subinhibitory concentrations of antibiotics
SMRT: single molecule, real-time
SNP: single nucleotide polymorphism
sp.: species
spp.: species
tRNA: transfer ribonucleic acid
UMN: University of Minnesota
UTR: untranslated region
