# Supplementary material for: Complete genome sequences of Streptomyces spp. isolated from disease-suppressive soils
Source: BMC Genomics. 2019 Dec 19;20:994. doi: 10.1186/s12864-019-6279-8 (PMC6923854; doi:10.1186/s12864-019-6279-8)
Supplement: Supplementary file 1 — Additional file 1: Table S1. Streptomyces sp. GS93–23, Table S2. Streptomyces sp. 3211–3,Table S3. Streptomyces sp. S3–4 gene clusters, Table S4. Cluster abundance for 125 Complete Streptomyces genomes, Figure S1. Indel comparison of Illumina polished vs. PacBio only assemblies. [file 12864_2019_6279_MOESM1_ESM.docx]

**Table S1. *Streptomyces sp.* GS93-23 gene clusters**

| Accession | Cluster | Predicted class | Start | End | Our Annotation |
| --- | --- | --- | --- | --- | --- |
| CP019457.1 | 1 | Lassopeptide | 195123 | 264181 |  |
|  | 2 | Thiopeptide-Lantipeptide | 439594* | 453423* | Cyclothiazomycin^1^ |
|  | 3 | Nrps-T1pks | 531139 | 609114 |  |
|  | 4 | Lantipeptide | 708680 | 731292 |  |
|  | 5 | Nrps | 908141 | 965139 |  |
|  | 6 | Bacteriocin-Cf_saccharide | 1059184 | 1101127 |  |
|  | 7 | T2pks | 1121146 | 1169639 |  |
|  | 8 | Nrps-Terpene-T1pks | 1505293* | 1593855* | Streptolydigin^2^ |
|  | 9 | Siderophore | 2053362* | 2058673* | Desferrioxamine B^3^ |
|  | 10 | Ectoine | 2142175* | 2145592* | Ectoine^4^ |
|  | 11 | Lassopeptide | 3346247 | 3368813 |  |
|  | 12 | T1pks-Cf_fatty_acid | 3696168 | 3743881 |  |
|  | 13 | Lantipeptide-Cf_fatty_acid | 4195732 | 4231551 |  |
|  | 14 | Terpene | 4654286 | 4676523 |  |
|  | 15 | Nrps | 4707417* | 4750551* | Mannopeptimycin^5^ |
|  | 16 | Siderophore | 6255261 | 6270018 |  |
|  | 17 | T3pks-Nrps | 6456534 | 6541453 |  |
|  | 18 | Butyrolactone | 6519962 | 6541453 |  |
|  | 19 | Bacteriocin | 6571918 | 6607981 |  |
|  | 20 | Terpene | 6988730 | 7035712 |  |
|  | 21 | Terpene | 7161797* | 7173404* | Hopene^6^ |
|  | 22 | Terpene | 7394231 | 7415328 |  |
|  | 23 | Melanin-Nrps-T1pks | 7578517 | 7647049 |  |
|  | 24 | Butyrolactone | 7845349 | 7866865 |  |
|  | 25 | Bacteriocin | 7991854 | 8035675 |  |
|  | 26 | Nrps-Cf_saccharide-Terpene | 7991854 | 8111863 |  |

**Table S2. *Streptomyces sp.* 3211-3 gene clusters**

| Accession | Cluster | Predicted class | Start | End | Annotation |
| --- | --- | --- | --- | --- | --- |
| CP020039.1 | 1 | Thiopeptide-Lantipeptide | 42452 | 86704 |  |
|  | 2 | Nrps-Ectoine-Cf_fatty_acid | 50628 | 162596 |  |
|  | 3 | Butyrolactone | 92595 | 162596 |  |
|  | 4 | Terpene | 164334 | 212168 |  |
|  | 5 | Bacteriocin | 490923 | 501096 |  |
|  | 6 | Ectoine | 650075* | 653448* | Ectoine^4^ |
|  | 7 | T2pks | 734087 | 776581 |  |
|  | 8 | Nrps | 1237647* | 1272586* | Tambromycin^7^ |
|  | 9 | Nrps-T1pks | 1365295* | 1385246* | Coelichelin^8^ |
|  | 10 | Butyrolactone | 1434091 | 1490762 |  |
|  | 11 | Cf_fatty_acid-Nrps-T1pks | 1434091 | 1545568 |  |
|  | 12 | Siderophore | 3095812* | 3103099* | Desferrioxamine B^3^ |
|  | 13 | Phosphonate-Ladderane-Cf_fatty_acid-T1pks | 3504301 | 3594577 |  |
|  | 14 | Nucleoside | 3533889 | 3597786 |  |
|  | 15 | Butyrolactone | 4231389 | 4272166 |  |
|  | 16 | Lantipeptide-Cf_fatty_acid-Arylpolyene | 5096360 | 5139726 |  |
|  | 17 | Siderophore | 6031316 | 6046390 |  |
|  | 18 | Nrps-T1pks | 6134059 | 6251759 |  |
|  | 19 | Bacteriocin | 6326616 | 6357733 |  |
|  | 20 | Terpene | 6468007 | 6536887 |  |
|  | 21 | T1pks-Cf_fatty_acid | 6488707 | 6536887 |  |
|  | 22 | Cf_saccharide-T1pks | 6488707 | 6599667 |  |
|  | 23 | Terpene | 6888835* | 6897732* | Hopene^6^ |
|  | 24 | T1pks-Otherks | 6942937 | 7014751 |  |
|  | 25 | Nrps | 7053970 | 7140641 |  |
|  | 26 | Nrps | 7234813 | 7285532 |  |
|  | 27 | Lantipeptide | 7399629 | 7428343 |  |
|  | 28 | Terpene | 7486707 | 7507804 |  |
|  | 29 | Lantipeptide | 7582565* | 7589118* | SapB^9^ |
|  | 30 | Terpene | 7646639* | 7648894* | 2-methylisoborneol^10^ |
|  | 31 | Terpene-Melanin | 7741551 | 7798205 |  |
|  | 32 | Siderophore | 7922498 | 7944290 |  |
|  | 33 | T3pks | 7965814 | 8015579 |  |
|  | 34 | Other | 8044240 | 8084893 |  |
|  | 35 | Other | 8071720 | 8115631 |  |
|  | 36 | Nrps | 8153326 | 8230599 |  |
| CP020040.1 | 37 | Lantipeptide | 275029 | 297872 |  |
|  | 38 | Nrps | 317542 | 369043 |  |

**Table S3. *Streptomyces sp.* S3-4 gene clusters**

| Accession | Cluster | Predicted class | Start | End | Our Annotation |
| --- | --- | --- | --- | --- | --- |
| CP020042.1 | 1 | Other | 293884 | 334687 |  |
|  | 2 | Other | 318352 | 362332 |  |
|  | 3 | Otherks-T1pks | 415409 | 467555 |  |
|  | 4 | Other | 460056 | 500778 |  |
|  | 5 | Nrps | 503717 | 547195 |  |
|  | 6 | Other | 599623 | 641515 |  |
|  | 7 | T2pks | 774236 | 816742 |  |
|  | 8 | T1pks | 810028 | 855541 |  |
|  | 9 | Thiopeptide-Lantipeptide | 1246647 | 1274503 |  |
|  | 10 | Nrps-T1pks-Cf_fatty_acid | 2543542 | 2608861 |  |
|  | 11 | Siderophore | 2890650* | 2897998* | Desferrioxamine B^3^ |
|  | 12 | Siderophore | 5727419 | 5741847 |  |
|  | 13 | Bacteriocin | 5989611 | 6000969 |  |
|  | 14 | Terpene | 6095043 | 6117265 |  |
|  | 15 | Nrps | 6256178 | 6324749 |  |
|  | 16 | Terpene | 6456916* | 6472585* | Hopene^6^ |
|  | 17 | Terpene | 6625217 | 6646326 |  |
|  | 18 | Nrps | 6755726* | 6783618* | Streptothricin^11^ |
|  | 19 | Terpene | 6634300* | 6636326* | 2-methylisoborneol^10^ |
|  | 20 | Melanin-Terpene | 6898352 | 6956176 |  |
|  | 21 | T3pks-Siderophore | 6999210 | 7042095 |  |
|  | 22 | Lantipeptide | 7197530* | 7203981* | SapB^9^ |
|  | 23 | Other | 7244572 | 7288504 |  |
|  | 24 | Bacteriocin | 7441958 | 7475954 |  |
| CP020043.1 | 25 | Nrps-T1pks-Melanin | 37745 | 168930 |  |
|  | 26 | Nrps | 157767 | 209377 |  |
|  | 27 | T1pks-Nrps-T2pks-Butyrolactone | 205255 | 348805 |  |
| CP020044.1 | 28 | T1pks | 1 | 188553 |  |

**Table S4. Cluster abundance for 125 Complete *Streptomyces* genomes**

| Organism | PATRIC Accession | Clusters | Contigs | Chromosomes | Plasmids | GC Content | Genome Size (bp) | CDS |
| --- | --- | --- | --- | --- | --- | --- | --- | --- |
|  |  |  |  |  |  |  |  |  |
| Streptomyces rapamycinicus NRRL 5491 | 1343740.8 | 49 | 1 | 1 | 0 | 70.60 | 12700734 | 10393 |
| Streptomyces bingchenggensis BCW-1 | 749414.3 | 48 | 1 | 1 | 0 | 70.75 | 11936683 | 10313 |
| Streptomyces sp. 11-1-2 | 1851167.4 | 47 | 2 | 1 | 1 | 70.85 | 11655206 | 10366 |
| Streptomyces autolyticus strain CGMCC0516 | 75293.3 | 46 | 8 | 1 | 7 | 71.21 | 10184660 | 8515 |
| Streptomyces hygroscopicus strain XM201 | 1912.5 | 46 | 1 | 1 | 0 | 70.75 | 12012215 | 10639 |
| Streptomyces sp. TLI_053 | 1855352.4 | 45 | 1 | 1 | 0 | 73.63 | 9900053 | 8757 |
| Streptomyces malaysiensis strain DSM 4137 | 92644.3 | 44 | 2 | 1 | 1 | 71.09 | 10744231 | 8968 |
| Streptomyces sp. PBH53 | 1577075.3 | 43 | 1 | 0 | 0 | 72.73 | 9153597 | 8401 |
| Streptomyces olivoreticuli strain ATCC 31159 | 284034.3 | 40 | 1 | 1 | 0 | 71.11 | 8809793 | 7923 |
| Streptomyces roseochromogenus subsp. oscitans DS 12.976 | 1352936.5 | 40 | 2 | 1 | 1 | 68.80 | 9782257 | 9636 |
| Streptomyces sp. CB01881 | 2078691.4 | 38 | 1 | 1 | 0 | 73.14 | 11344947 | 9001 |
| Streptomyces sp. 769 | 1262452.3 | 38 | 2 | 1 | 1 | 71.60 | 10338286 | 9177 |
| Streptomyces hygroscopicus subsp. jinggangensis TL01 | 1203460.3 | 38 | 3 | 1 | 2 | 42.71 | 10077952 | 9146 |
| Streptomyces albireticuli strain SMD11 | 1940.3 | 38 | 1 | 1 | 0 | 72.82 | 8144417 | 7301 |
| Streptomyces hygroscopicus subsp. jinggangensis 5008 | 1133850.2 | 38 | 3 | 1 | 2 | 71.84 | 10383684 | 9901 |
| Streptomyces sp. CFMR 7 | 1649184.3 | 38 | 2 | 1 | 1 | 72.03 | 8307279 | 7407 |
| Streptomyces clavuligerus strain F1D-5 | 1901.15 | 38 | 3 | 1 | 2 | 72.53 | 8059125 | 7323 |
| Streptomyces sp. ICC1 | 2099583.3 | 37 | 1 | 1 | 0 | 72.02 | 9034319 | 8909 |
| Streptomyces albulus ZPM | 1434306.3 | 37 | 1 | 1 | 0 | 72.13 | 9784577 | 9148 |
| Streptomyces albolongus strain YIM 101047 | 68173.3 | 37 | 1 | 1 | 0 | 71.98 | 8027788 | 7261 |
| Streptomyces avermitilis MA-4680 | 227882.9 | 37 | 2 | 1 | 1 | 70.70 | 9119895 | 8106 |
| Streptomyces armeniacus strain ATCC 15676 | 83291.4 | 36 | 1 | 1 | 0 | 72.37 | 8083249 | 7273 |
| Streptomyces sp. Sge12 strain Sge12 | 1972846.3 | 36 | 2 | 1 | 1 | 72.17 | 8110698 | 7511 |
| Streptomyces silaceus strain ACCC40021 | 545123.5 | 36 | 1 | 1 | 0 | 72.09 | 8625867 | 7813 |
| Streptomyces griseus subsp. griseus NBRC 13350 | 455632.4 | 36 | 1 | 1 | 0 | 72.20 | 8545929 | 7294 |
| Streptomyces albulus strain NK660 | 68570.5 | 35 | 2 | 1 | 1 | 72.32 | 9372401 | 8793 |
| Streptomyces sp. ICC4 | 2099584.3 | 35 | 1 | 1 | 0 | 72.03 | 9010404 | 8878 |
| Streptomyces fradiae strain NKZ-259 | 1906.17 | 35 | 1 | 1 | 0 | 72.13 | 8081458 | 7431 |
| Streptomyces lydicus A02 | 1403539.3 | 35 | 1 | 1 | 0 | 70.70 | 9300149 | 8888 |
| Streptomyces sp. P3 | 2135430.3 | 35 | 1 | 1 | 0 | 71.37 | 9851971 | 9315 |
| Streptomyces albus DSM 41398 | 1888.4 | 35 | 1 | 1 | 0 | 72.64 | 8384669 | 6923 |
| Streptomyces luteoverticillatus strain CGMCC 15060 | 66425.3 | 35 | 1 | 1 | 0 | 72.05 | 7367863 | 6832 |
| Streptomyces formicae strain KY5 | 1616117.3 | 34 | 1 | 1 | 0 | 71.38 | 9611874 | 8393 |
| Streptomyces albus strain BK3-25 | 1888.11 | 34 | 1 | 1 | 0 | 72.64 | 8308430 | 7171 |
| Streptomyces lincolnensis strain NRRL 2936 | 1915.4 | 34 | 1 | 1 | 0 | 71.01 | 10319054 | 9514 |
| Streptomyces hygroscopicus subsp. limoneus KCTC 1717 | 264445.3 | 34 | 2 | 2 | 0 | 71.96 | 10537932 | 9983 |
| Streptomyces anulatus strain ATCC 11523 | 1892.7 | 34 | 2 | 1 | 1 | 71.72 | 8847108 | 8036 |
| Streptomyces fulvissimus DSM 40593 | 1303692.3 | 34 | 1 | 1 | 0 | 71.50 | 7905758 | 7081 |
| Streptomyces clavuligerus strain F613-1 | 1901.9 | 34 | 2 | 1 | 1 | 72.60 | 7590758 | 6641 |
| Streptomyces sp. NEAU-S7GS2 | 2202000.4 | 34 | 2 | 1 | 1 | 70.78 | 9687439 | 9008 |
| Streptomyces lydicus strain WYEC 108 | 47763.27 | 34 | 1 | 1 | 0 | 70.80 | 9125666 | 8388 |
| Streptomyces pactum strain KLBMP 5084 | 68249.6 | 34 | 1 | 1 | 0 | 72.41 | 8180260 | 7562 |
| Streptomyces sp. CdTB01 | 1725411.3 | 33 | 2 | 1 | 1 | 71.53 | 10191567 | 9718 |
| Streptomyces sp. 2323.1 | 1938841.3 | 33 | 1 | 1 | 0 | 71.19 | 8212455 | 7369 |
| Streptomyces puniciscabiei strain TW1S1 | 164348.7 | 33 | 1 | 1 | 0 | 71.09 | 9698948 | 9166 |
| Streptomyces sp. S8 strain S8 | 1837283.3 | 33 | 2 | 1 | 1 | 72.29 | 7601864 | 6837 |
| Streptomyces griseoviridis strain F1-27 | 45398.5 | 33 | 1 | 1 | 0 | 72.38 | 8963414 | 8148 |
| Streptomyces sp. Mg1 | 465541.12 | 32 | 4 | 1 | 3 | 72.13 | 8716193 | 8218 |
| Streptomyces collinus Tu 365 | 1214242.5 | 32 | 3 | 1 | 2 | 72.55 | 8377286 | 7336 |
| Streptomyces cyaneogriseus NMWT 1 | 477245.3 | 32 | 1 | 1 | 0 | 72.86 | 7762396 | 6922 |
| Streptomyces bacillaris strain ATCC 15855 | 68179.3 | 32 | 1 | 1 | 0 | 71.95 | 7888441 | 7125 |
| Streptomyces sp. HNM0039 | 2174846.3 | 31 | 1 | 1 | 0 | 72.46 | 7289495 | 6755 |
| Streptomyces cavourensis strain TJ430 | 67258.5 | 31 | 1 | 1 | 0 | 72.12 | 7613757 | 6798 |
| Streptomyces cavourensis strain 1AS2a | 67258.4 | 31 | 1 | 1 | 0 | 72.13 | 7600475 | 6788 |
| Streptomyces sp. ZFG47 | 2184053.3 | 31 | 2 | 1 | 1 | 70.76 | 10239272 | 9684 |
| Streptomyces davawensis JCM 4913 | 1214101.3 | 31 | 2 | 1 | 1 | 70.59 | 9555950 | 8696 |
| Streptomyces chartreusis NRRL 3882 strain NRRL3882 | 1079985.1 | 31 | 1 | 1 | 0 | 71.23 | 8983317 | 8293 |
| Streptomyces sp. Go-475 | 2072505.3 | 31 | 1 | 1 | 0 | 71.96 | 8570609 | 7865 |
| Streptomyces leeuwenhoekii NRRL B-24963 | 1437453.6 | 31 | 3 | 1 | 2 | 72.68 | 8122491 | 7432 |
| Streptomyces violaceoruber strain S21 | 1935.7 | 31 | 1 | 1 | 0 | 72.65 | 7916045 | 7235 |
| Streptomyces incarnatus strain NRRL8089 | 665007.5 | 30 | 4 | 0 | 3 | 71.48 | 8897465 | 8452 |
| Streptomyces venezuelae | 54571.11 | 30 | 1 | 1 | 0 | 71.75 | 9034396 | 8487 |
| Streptomyces niveus NCIMB 11891 | 1352941.4 | 30 | 5 | 1 | 4 | 69.44 | 8726876 | 8170 |
| Streptomyces lincolnensis strain LC-G | 1915.7 | 30 | 1 | 1 | 0 | 71.06 | 9513637 | 8737 |
| Streptomyces sp. Tue 6075 | 1661694.4 | 30 | 1 | 1 | 0 | 71.57 | 7931832 | 7175 |
| Streptomyces vietnamensis GIM4.0001 | 362257.4 | 30 | 2 | 1 | 1 | 71.99 | 9153777 | 8292 |
| Streptomyces venezuelae strain NRRL B-65442 | 54571.16 | 30 | 2 | 0 | 1 | 72.42 | 8380320 | 7771 |
| Streptomyces sp. GSSD-12 | 2282738.3 | 30 | 1 | 1 | 0 | 71.19 | 8454852 | 7430 |
| Streptomyces venezuelae ATCC 10712 | 953739.5 | 30 | 1 | 1 | 0 | 72.40 | 8226158 | 7409 |
| Streptomyces gilvosporeus strain F607 | 553510.3 | 29 | 1 | 1 | 0 | 70.95 | 8482298 | 7969 |
| Streptomyces coelicolor A3(2) | 100226.15 | 29 | 3 | 1 | 2 | 72.00 | 9054847 | 8325 |
| Streptomyces bottropensis ATCC 25435 | 1054862.11 | 29 | 1 | 0 | 0 | 71.19 | 8955726 | 8324 |
| Streptomyces venezuelae strain ATCC 15439 | 54571.1 | 28 | 1 | 1 | 0 | 71.74 | 9054831 | 8457 |
| Streptomyces niveus strain SCSIO 3406 | 193462.5 | 28 | 1 | 1 | 0 | 70.46 | 7990492 | 7301 |
| Streptomyces laurentii strain ATCC 31255 | 39478.4 | 28 | 1 | 1 | 0 | 72.**30** | 8032664 | 7709 |
| Streptomyces lunaelactis strain MM109 | 1535768.3 | 28 | 3 | 1 | 2 | 69.75 | 8570191 | 8269 |
| Streptomyces sp. CNQ-509 | 444103.5 | 28 | 1 | 1 | 0 | 73.07 | 8039333 | 7243 |
| Streptomyces sp. SCSIO 03032 strain SCSIO 03032 | 1109743.5 | 28 | 1 | 1 | 0 | 73.52 | 6287975 | 5677 |
| Streptomyces sp. PAMC26508 | 1265601.4 | 28 | 2 | 1 | 1 | 71.06 | 7630245 | 6806 |
| Streptomyces sp. S063 | 2005885.3 | 28 | 1 | 1 | 0 | 71.49 | 7614683 | 7330 |
| Streptomyces spongiicola strain HNM0071 | 1690221.3 | 27 | 1 | 1 | 0 | 72.45 | 7180417 | 6703 |
| Streptomyces globisporus strain TFH56 | 1908.1 | 27 | 3 | 1 | 2 | 71.54 | 7666521 | 7248 |
| Streptomyces sp. fd1-xmd strain fd1-xmd | 1812480.3 | 27 | 1 | 1 | 0 | 72.51 | 7929999 | 7460 |
| Streptomyces sp. S10(2016) | 1783515.3 | 27 | 1 | 1 | 0 | 71.26 | 9083372 | 8480 |
| Streptomyces aureofaciens strain DM-1 | 1894.18 | 27 | 2 | 1 | 1 | 72.57 | 7076402 | 6652 |
| Streptomyces sp. TN58 | 234612.4 | 27 | 1 | 1 | 0 | 72.30 | 7585034 | 7194 |
| Streptomyces lydicus strain GS93 | 47763.1 | 27 | 1 | 1 | 0 | 72.01 | 8243179 | 7497 |
| Streptomyces lividans TK24 | 457428.16 | 27 | 1 | 1 | 0 | 72.24 | 8345283 | 7749 |
| Streptomyces flavogriseus ATCC 33331 | 591167.6 | 27 | 3 | 1 | 2 | 71.00 | 7656104 | 6866 |
| Streptomyces alboflavus strain MDJK44 | 67267.5 | 27 | 1 | 1 | 0 | 72.09 | 9622415 | 9185 |
| Streptomyces sp. MOE7 strain MOE7 | 1961713.3 | 26 | 1 | 1 | 0 | 71.99 | 8399509 | 7802 |
| Streptomyces sp. CMB-StM0423 | 2059884.3 | 26 | 1 | 1 | 0 | 73.14 | 8029398 | 7153 |
| Streptomyces globosus strain LZH-48 strain soil | 68209.3 | 26 | 3 | 1 | 2 | 73.65 | 7535750 | 6974 |
| Streptomyces sp. 3124.6 strain 3124.6 | 1882757.3 | 26 | 1 | 1 | 0 | 70.67 | 9375551 | 8748 |
| Streptomyces lydicus strain 103 | 47763.9 | 26 | 1 | 1 | 0 | 72.22 | 8201357 | 7502 |
| Streptomyces ambofaciens ATCC 23877 | 278992.5 | 26 | 2 | 1 | 1 | 72.19 | 8393598 | 7953 |
| Streptomyces sp. PVA 94-07 | 1223307.4 | 26 | 3 | 1 | 2 | 73.05 | 7106149 | 6359 |
| Streptomyces sp. GBA 94-10 | 1218177.5 | 26 | 2 | 1 | 1 | 72.96 | 7224475 | 6566 |
| Streptomyces sp. 2114.2 | 1881022.3 | 26 | 1 | 1 | 0 | 72.11 | 8620263 | 8008 |
| Streptomyces atratus strain SCSIO_ZH16 | 1893.7 | 26 | 1 | 1 | 0 | 69.59 | 9641288 | 9294 |
| Streptomyces sp. CC0208 | 2306165.3 | 25 | 1 | 1 | 0 | 70.59 | 9320089 | 8728 |
| Streptomyces glaucescens GLA.O | 1907.4 | 25 | 2 | 1 | 1 | 72.91 | 7623774 | 6719 |
| Streptomyces ambofaciens strain DSM 40697 | 1889.1 | 25 | 1 | 1 | 0 | 72.33 | 8137876 | 7413 |
| Streptomyces griseorubiginosus strain 3E-1 | 67304.8 | 25 | 1 | 1 | 0 | 70.94 | 9512378 | 8665 |
| Streptomyces sp. WAC00288 | 2094021.4 | 24 | 5 | 1 | 4 | 72.73 | 7852859 | 7532 |
| Streptomyces sp. XZHG99 | 2049881.3 | 24 | 3 | 1 | 2 | 69.92 | 8710171 | 8663 |
| Streptomyces rubrolavendulae strain MJM4426 | 285473.5 | 24 | 1 | 1 | 0 | 74.78 | 6543262 | 5844 |
| Streptomyces sp. CCM_MD2014 | 1561022.5 | 24 | 1 | 1 | 0 | 72.13 | 8274043 | 7689 |
| Streptomyces actuosus strain ATCC 25421 | 1885.4 | 23 | 1 | 1 | 0 | 72.53 | 8145579 | 7618 |
| Streptomyces sp. SirexAA-E | 862751.12 | 22 | 1 | 1 | 0 | 71.75 | 7414440 | 6808 |
| Streptomyces sp. CB09001 | 2083284.3 | 22 | 1 | 1 | 0 | 71.95 | 7787608 | 7303 |
| Streptomyces parvulus strain 2297 | 146923.5 | 22 | 2 | 1 | 1 | 72.73 | 7766531 | 7056 |
| Streptomyces peucetius subsp. caesius ATCC 27952 | 316280.3 | 22 | 1 | 1 | 0 | 70.59 | 8023114 | 7759 |
| Streptomyces albus J1074 | 457425.27 | 22 | 1 | 1 | 0 | 73.32 | 6841649 | 6120 |
| Streptomyces koyangensis strain VK-A60T | 188770.3 | 21 | 1 | 1 | 0 | 73.03 | 7220839 | 6695 |
| Streptomyces sp. FR-008 | 206662.6 | 21 | 3 | 1 | 2 | 73.32 | 7258031 | 6897 |
| Streptomyces sp. 452 | 1827580.3 | 21 | 1 | 1 | 0 | 71.91 | 7641029 | 7171 |
| Streptomyces sampsonii strain KJ40 | 42239.3 | 21 | 1 | 1 | 0 | 73.39 | 7070328 | 6359 |
| Streptomyces albus strain SM254 | 1888.8 | 21 | 1 | 1 | 0 | 73.34 | 7170504 | 6473 |
| Streptomyces pristinaespiralis strain HCCB 10218 | 38300.4 | 21 | 1 | 1 | 0 | 71.54 | 8532592 | 7700 |
| Streptomyces sp. ETH9427 | 2211357.3 | 20 | 3 | 1 | 2 | 72.00 | 7978031 | 7803 |
| Streptomyces xiamenensis strain MCCC 1A01550 | 408015.6 | 20 | 1 | 1 | 0 | 72.02 | 5961401 | 5556 |
| Streptomyces nodosus ATCC 14899 | 40318.3 | 20 | 1 | 1 | 0 | 70.80 | 7714110 | 6875 |
| Streptomyces sp. 4F | 1751294.3 | 19 | 1 | 1 | 0 | 72.28 | 8047771 | 7510 |
| Streptomyces sp. CLI2509 | 1984801.3 | 18 | 2 | 1 | 1 | 73.30 | 7232701 | 6733 |

**Figure S1. Indel comparison of Illumina polished vs. PacBio only assemblies**

**
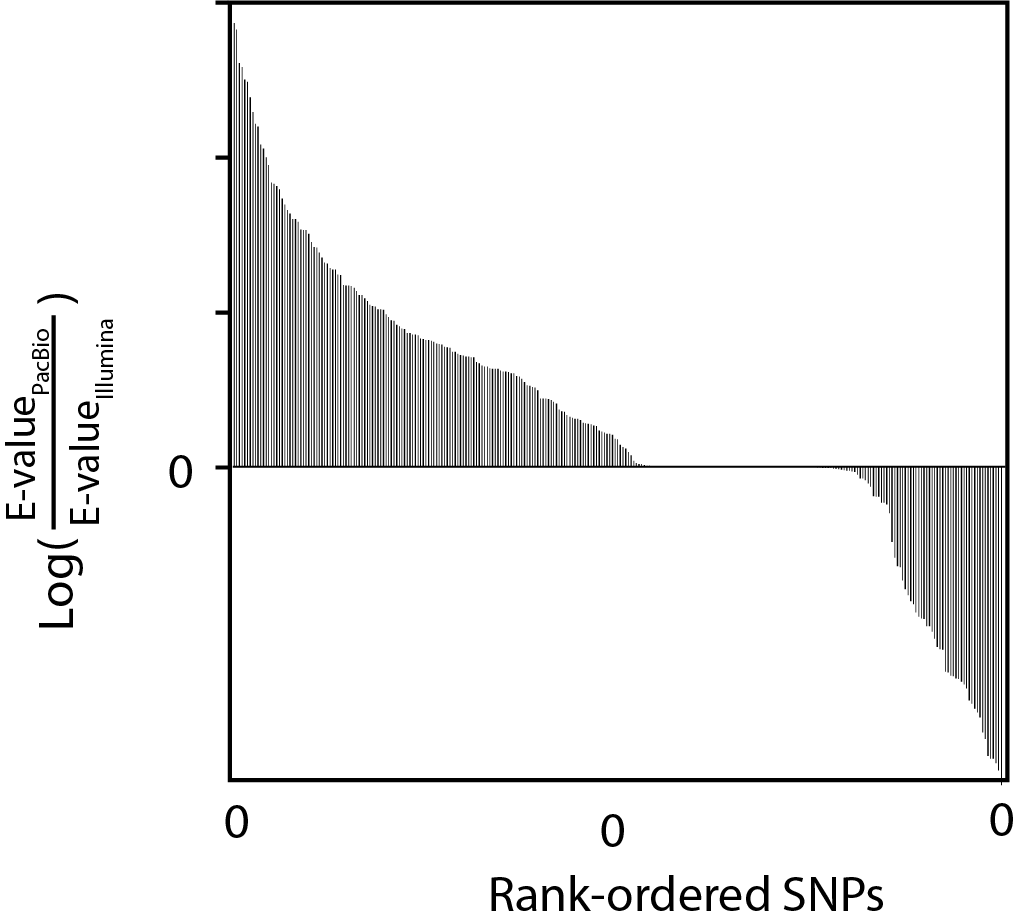
**

**Supplementary Methods**

**Cluster abundance comparison**

Unannotated sequences for 125 complete status *Streptomyces* genomes were downloaded from the PATRIC database^12^. Sequences were annotated with antiSMASH 4.1. Detection of putative clusters was disabled. A Python script was used to count the number of cluster features in the resulting genbank files. The cluster abundance data was combined with genome statistics from the PATRIC database including contig, chromosome, and plasmid counts, GC content, genome size, and CDS counts.

**Signaling potential analysis**

MultiGeneBlast^13^ was used to query the 496 genomes from the phylogenetic analysis dataset. Query sequences were ScbA and AfsR homologue amino acid sequences from γ-butyrolactone BGCs from 3211-3, S3-4, or GS93-23. Genome IDs were sorted in descending order of clusterBLAST score from 3211-3. Identity scores from the phylogenetic analysis were plotted against clusterBLAST scores.

**REFERENCES**

1. Wang, J. *et al.* Identification and analysis of the biosynthetic gene cluster encoding the thiopeptide antibiotic cyclothiazomycin in Streptomyces hygroscopicus 10-22. *Appl. Environ. Microbiol.* **76**, 2335–2344 (2010).

2. Olano, C. *et al.* Deciphering biosynthesis of the RNA polymerase inhibitor streptolydigin and generation of glycosylated derivatives. *Chem. Biol.* **16**, 1031–1044 (2009).

3. Barona-Gomez, F., Wong, U., Giannakopulos, A. E., Derrick, P. J. & Challis, G. L. Identification of a cluster of genes that directs desferrioxamine biosynthesis in Streptomyces coelicolor M145. *J. Am. Chem. Soc.* **126**, 16282–16283 (2004).

4. Ofer, N. *et al.* Ectoine biosynthesis in Mycobacterium smegmatis. *Appl. Environ. Microbiol.* **78**, 7483–7486 (2012).

5. Magarvey, N. A., Haltli, B., He, M., Greenstein, M. & Hucul, J. A. Biosynthetic pathway for mannopeptimycins, lipoglycopeptide antibiotics active against drug-resistant gram-positive pathogens. *Antimicrob. Agents Chemother.* **50**, 2167–2177 (2006).

6. Siedenburg, G. & Jendrossek, D. Squalene-hopene cyclases. *Applied and Environmental Microbiology* **77**, 3905–3915 (2011).

7. Goering, A. W. *et al.* Metabologenomics: Correlation of Microbial Gene Clusters with Metabolites Drives Discovery of a Nonribosomal Peptide with an Unusual Amino Acid Monomer. *ACS Cent. Sci.* **2**, 99–108 (2016).

8. Lautru, S., Deeth, R. J., Bailey, L. M. & Challis, G. L. Discovery of a new peptide natural product by Streptomyces coelicolor genome mining. *Nat. Chem. Biol.* **1**, 265–269 (2005).

9. Kodani, S. *et al.* The SapB morphogen is a lantibiotic-like peptide derived from the product of the developmental gene ramS in Streptomyces coelicolor. *Proc. Natl. Acad. Sci. U. S. A.* **101**, 11448–11453 (2004).

10. Wang, C. M. & Cane, D. E. Biochemistry and molecular genetics of the biosynthesis of the earthy odorant methylisoborneol in Streptomyces coelicolor. *J. Am. Chem. Soc.* **130**, 8908–8909 (2008).

11. Maruyama, C. *et al.* A stand-alone adenylation domain forms amide bonds in streptothricin biosynthesis. *Nat. Chem. Biol.* **8**, 791–797 (2012).

12. Wattam, A. R. *et al.* Improvements to PATRIC, the all-bacterial Bioinformatics Database and Analysis Resource Center. *Nucleic Acids Res.* **45**, D535–D542 (2017).

13. Medema, M. H., Takano, E. & Breitling, R. Detecting Sequence Homology at the Gene Cluster Level with MultiGeneBlast. *Mol. Biol. Evol.* **30**, 1218–1223 (2013).
